# Probing rate of YMDD motif mutant in lamivudine treatment of Iranian patients with chronic hepatitis B virus infection

**DOI:** 10.4103/0973-6247.75982

**Published:** 2011-01

**Authors:** Freshte Ghandehari, Abasali Pourazar, Mehrnaz Shanehsaz Zadeh, Nahid Tajedin

**Affiliations:** *Department of Microbiology and Genetics, Islamic Azad University Fellavarjan Branch, Isfahan, Iran*; 1 *Department of Immunology, Isfahan University of Medical Sciences, Isfahan, Iran*; 2 *Medical Biotechnologist, Park Pathology Lab, Isfahan, Iran*

**Keywords:** Chronic hepatitis B, lamivudine, PCR flash, RFLP, YMDD motif mutants

## Abstract

**Background:**

Lamivudine is an analog nucleoside used for the treatment of chronic hepatitis B virus (CHV) infection. Studies showed that prolonged therapy could induce lamivudine resistance hepatitis B virus (HBV) variants (YMDD motif). In this study, the rate of YMDD motif mutants is determined in lamivudine-treated CHV patients in Iran.

**Materials and Methods:**

Thirty-three serum specimens of patients with chronic hepatitis who had been treated with lamivudine were included in the study. Serum samples of patients were tested by PCR flash and RFLP as well as tested for HBeAg, HBsAg, and liver enzymes (ALT and AST).

**Results:**

Out of the 33 patients enrolled in this study, 82% were male and 18% female, respectively. Mean ALT levels were between 20 and 100 IU/1. HBeAg was positive in 76% of the patients whereas HBs was positive only in 61% of the patients. Furthermore, in 28 patients liver biopsy grade was between 2 and 17 having the stage of 1–6. Moreover, HBsAg negative and HBeAg positive were observed in 30% of the patients.

**Conclusion:**

During therapy, it was found that patients with lamivudine incidence YMDD mutation were approximately 14%. The ALT levels were also reduced in these patients. This study revealed that there was a significant difference between HBeAg, grade, and YMDD mutation whereas no significant different was observed between HBsAg and HBV DNA PCR. Conclusively, it was found that a significant difference exists between YMDD mutation and lamivudine therapy (17% of patients were HBsAg negative and PCR positive).

## Introduction

Hepatitis is an inflammation of liver which could be induced by several agents such as chemical agents, drugs, and viruses.[1] Viral hepatitis refers specifically to a primary infection of the liver by one of the seven subtypes of hepatitis viruses that although are etiologically associated, but are different as types A, B, C, D, E, F, and G. The clinical manifestations of almost all these types are similar.[2] Hepatitis B virus (HBV) is a blood-borne pathogen and is a member of the hepadnaviridae, and has a relaxed circular partially double-stranded DNA genome, HBV can cause serious liver diseases and replication via reverse transcription using its own RNA-dependent DNA polymerase.[3] HBV causes an acute and chronic hepatitis. Chronic hepatitis B (CHV) remains a major public health problem affecting more than 350 million people worldwide. The CHV virus may develop into cirrhosis, liver failure, or hepatocellular carcinoma.[4] Lamivudine is an oral nucleoside (B-L, 2-3-dioxy-3-thiacytidine), nontoxic, and inhibitor of RNA-dependent DAN polymerase.[5] Treatment with oral lamivudine is beneficial to both HBeAg positive and negative CHV. Lamivudine can also reduce hepatic necroinflammatory activity serum HBV DNA levels and normalize alanine aminotransferase (ALT) levels.[5,6,12] Although lamivudine can delay clinical progression in patients, long-term monotherapy can lead to lamivudine-resistant mutants. This mutation of the tyrosine–methionine–aspartate (YMDD) motif in the C domain of the HBV DNA polymerase gene, the amino acid substitution from methionine to valine (YVDD) or an isoleucine (YIDD) at amino acid 552 of the YMDD motif of the polymerse gene is the main mutation responsible for resistance to the lamivudine treatment.[6] Recent studies suggest that with the occurrence of lamivudine resistance, there is a need for a combinational antiviral therapy. This shall prevent progressive liver diseases.[4] In this study, mutation in the HBV DNA polymerase gene was determined utilizing polymerase chain reaction (PCR) as well as examination of codon position 552 of the polymerase gene by the restriction fragment length polymorphism (RFLP).

## Materials and Methods

Thirty-three serum specimens collected from patients with chronic hepatitis who took lamivudine orally 100 mg every day for 12–48 months. They did not receive any other antiviral therapy during the study period. Later on, all cases were diagnosed according to the tests performed previously. The HBe and HBsAg levels in the patient sera were measured with enzyme linked immuno sorbent assay (ELISA).

Out of 33 patients included, they were found 20 with HBsAg and 25 with HBeAg positive. Simultaneously, liver enzyme (ALT and AST) was also determined in all of them. The DAN was detected in 20 patients with the PCR flash using the Biogen kit.

### Extraction of DAN from Patient Sera

DAN was extracted from 100 μl serum incubated with proteinase K in lysis buffer at 65 °C for 20 min followed by phenol/chloroform extraction and ethanol precipitation. Finally, the pellet was dissolved in 50 μl sterile DNAase free water and stored at −20 °C which subsequently further tested.

### HBV DNA detection

The isolated HBV DNA was amplified by PCR using primers: F1(5´-CACTGTTTGGCTTTCAGTCAT-3´), F3 (5´-GTGGGCCTCAGTC CGTTTCTC-3´) and B2(5´- GTTCAAATGTATACGAAA TGTATACGCAAAG-3´). The primers were designed to amplify the particular sequence coding for the YMDD motif of the viral polymerase in order to examine the 528 codon, primer pairs f3 and B2 were used for PCR amplification. The variation at the 528 codon associated with lamivudine resistance *in vitro*, from leucine (C/TTG) to methionine (ATG) created an N1aIII restriction to examine the 552 codon by RFLP analysis. The primer pairs of F1 and B2 were used for amplification. For detecting variation at codon 552 in the HBV polymerase, the YMDD nucleotide sequence TATATGCATGAT was used. The creation of an NdeI site occurs only if the template HBV has a wide-type DNA sequence and is absent if the template HBV mutated NdeI had sited (CA↓TATG) once the HBVDNA template was of the wide-type sequence (methionine ATG). However, the presence of valine or isoleucine variations at 552 created a new restriction site, N1aIII (CATG↓), allowing the determination of the presence YMDD mutation. HBV DNA extracted from patient sera was amplified by PCR in a final volume of 25 μl containing 20 mmol Tris–HCL (pH = 8.3), 18 mmol NaCl, 2 mmol MgCl_2_, 50 M KCl, 0.2 μmol of each primer, 1.5 μl of Ampli Taq polymerase, 0.2 mmol dNTPS, and 5 μl DNA.

**Figure 1:**
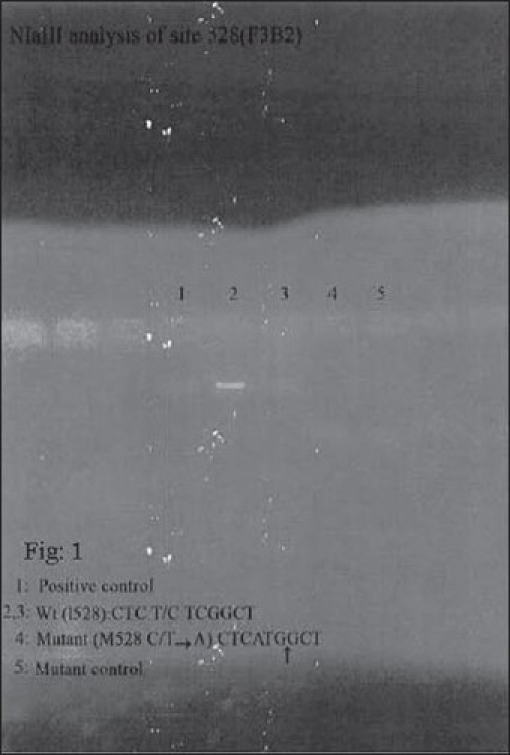
NIaIII analysis of site 528(F3B2)

**Figure 2:**
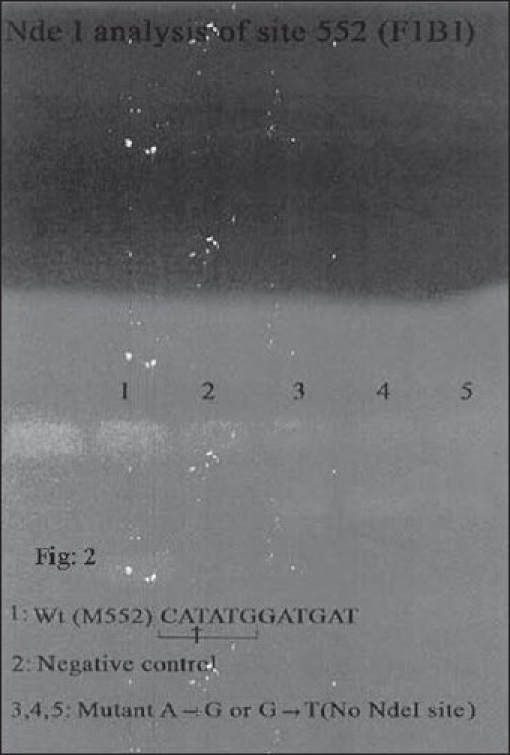
NdeI analysis of site 552(F1B1)

Thermocycler conditions for amplification were 35 cycles, at 95 °C for 30 s, 55 °C for 45 s, and 72 °C for 30 s followed by a hold at 8 °C. A 5 μl aliquot of each PCR product was digested with NdeI or N1aIII for 1 h at 37 °C in separate reactions. The resulting DNA fragments were determined by 3% agarose gel electrophoresis. The RFLP results become visible under UV light after staining with ethidium bromide [Figures 1 and 2].

### Statistical Methods

The Chi-square test was used for statistical analysis. A *P* value of >0.05 was considered as statistically significant.

## Results

A total of 33 patients were enrolled in this study. Of them 82% were male and 18% were female and aged between 16 and 62 years. CHV diagnosed in patients was on the basis of HBs and HBeAg, and PCR. The patients’ ALT levels were between 20 and 100 IU/1. HBsAg was found positive in 61% of the patients, and HBeAg in 76% of the patients among 28 patients; and in 17% of patients with HBs negative, PCR was positive. The diagnosis of CHV was on the basis of liver biopsy. The liver biopsy grade was between 2 and 17 having the stage between 1 and 6 according to the Knodell Necroinflammatory Score System. PCR was found to be positive in 61% of patients. However, YMDD motif mutations were detected in four patients having HBs and PCR negative (12%).

## Discussion

Lamivudine is an analog nucleoside with a potential inhibitor of HBV[5] and is taken orally at a dosage of 100 mg daily.[7] Prolonged therapy of chronic hepatitis with lamivudine can develop lamivudine-resistant HBV variants.[6] Yuen *et al*. (2003) reported that the incidence rate of YMDD mutation was 22% in 283 patients treated with lamivudine[15] and Li *et al*. (2006) showed that YMDD mutation rates were 16–32%, 47–56%, and 62–75% in 142 patients administered with lamivudine for 1, 2, and 3 years.[9] Also Masaadeh *et al*. (2008) suggested 31% rate YMDD mutation in 107 patients.[14] In addition, according to Hoonkoop *et al* also observed mutation in 4 out of 81 treated patients with CHV[10] and also lamivudine-resistant can develop in 14–32% of patients after 1 year.[8] In a study from Asia (2001), YMDD mutations were 15, 38, and 53% after lamivudine treatment for 1, 2, and 3 years, respectively.[17] Studies showed that although lamivudine may reduce serum HBV DAN levels and normalize alanine aminotransferase (ALT) levels and liver necroinflammatory activity in patients,[16] but the greatest draw back with lamivudine pretreatment is the emergency of YMDD mutant with concomitant rise in ALT, HBV DNA and worsening histology in some patients.[8,13,16] Liaw *et al*. (2002) reported emergency YMDD mutation with an incidence of 38% and 67% after 2 and 4 years of lamivudine therapy.[11] It was also found that ALT and HBV DNA elevations to be at lower median levels than their base lines during the lamivudine therapy.[7,11] Yun-fan *et al*. (1999) recorded that patients with YMDD mutation showed lower median serum HBV DNA and ALT levels during the lamivudine therapy. Acute exacerbation occurred in 41% after the incidence of YMDD mutation.[13] Factors such as age, gender, and disease status had no effect on the rate of lamivudine-resistant mutations.[8] However, factors such as status of HBV antigen was related to mutation. Ghany *et al*.[5] found that in 16–18% of patients, HBeAg seroconversion was observed; also Kobayashi *et al*. showed that in all patients with YMDD anti-HBe was found to be positive.[8] A study conducted in China had shown that the incidence rate of YMDD mutation was 26.1% in negative HBeAg and positive anti-HBe and 27.1% in patients with positive HBeAg.[12] Yun-Fan *et al*. (1995) reported 75% HBeAg seroconversion in 12 patients with the YMDD mutant.[13] Yuen *et al*. reported that ALT, HBV DNA levels induced in patients with mutation after the treatment and also showed a higher rate of HBeAg seroconversion.[15] It was shown in our study that the rate of lamivudine resistance is 14% in patients with CHV infection after 12–48 months of lamivudine therapy. These results are similar to studies of Pie *et al* and Li *et al*.[4,9] The patients also showed serum alanine amino transferase (ALT) at the lower level than the base line (range, 20–100) after therapy. In addition, it was found that the patients with the YMDD mutant were HBV-DNA and HBsAg negative and median ALT level was 42 (range, 20-60), but these patients were HBeAg positive. These results accord with other studies.[4,7,11] According to the Chi-square test, there is a significant difference between HBeAg, grade, and YMDD mutation, but the significant difference was not observed between HBsAg and HBV-DNA PCR (17% of patients were HBs Ag negative and PCR positive). To sum up, it can be concluded that significant difference exists between YMDD mutation and lamivudine therapy. Therefore, it is suggested that upon occurrence of YMDD mutation an analog nucleotide (e.g., adefovir) was used as a supplement. There is also no benefit in continuing lamivudine therapy in patients with YMDD mutation.
